# SDF-1/CXCR4 Axis Promotes MSCs to Repair Liver Injury Partially through Trans-Differentiation and Fusion with Hepatocytes

**DOI:** 10.1155/2015/960387

**Published:** 2015-08-02

**Authors:** Ning-Bo Hao, Chang-Zhu Li, Mu-Han Lü, Bo Tang, Su-Min Wang, Yu-Yun Wu, Guang-Ping Liang, Shi-Ming Yang

**Affiliations:** ^1^Department of Gastroenterology, Xinqiao Hospital, Third Military Medical University, Chongqing 400037, China; ^2^Institute of Burn Research, Southwest Hospital, Third Military Medical University, Chongqing 400038, China

## Abstract

MSCs have become a popular target for developing end-stage liver therapies. In this study, two models of bone marrow chimeric mice were used to construct the liver failure models. Then it was found that MSCs can transdifferentiate into hepatocyte-like cells and these hepatocyte-like cells can significantly express albumin. Furthermore it was also found that MSCs can fuse with the hepatocytes and these cells had the proliferation activity. However, the percentage of transdifferentiation was significantly higher than fusion. So it was considered that MSCs which transdifferentiated into hepatocyte-likes cells played important roles for repairing the injuring liver function.

## 1. Introduction

End-stage liver disease (ELSD) is an irreversible condition that leads to the imminent complete failure of the liver [1]. It is often a consequence of chronic liver diseases and is one of the most common causes of death in China. ESLD may be the final stage of many liver diseases. Cirrhosis, viral hepatitis, genetic disorders, autoimmune disorder, toxins, and drugs are all factors that cause ESLD and liver failure [2]. Studies have shown that patients with ESLD are at high mortality risk because of a high incidence of concomitant infection and renal and respiratory failure [2]. The effective therapy for patients with ESLD is liver transplantation [3]. However, many drawbacks such as the relative shortage of donors, operative risk, posttransplant rejection, recidivism of the preexisting liver disease, and high cost limit this technology [4]. Therefore, the exploration of new therapeutic approaches is necessary.

In recent years, bone marrow-derived mesenchymal stem cells (MSCs) have been a popular topic in regenerative medicine and have generated a great amount of enthusiasm as a therapeutic paradigm for a series of diseases. MSCs are a subset of plastic adherent nonhematopoietic stem cells and are characterized by their ability for self-renewal and differentiation into multiple cell types, such as osteoblasts, adipocytes, and chondrocytes [5]. As early as in 1968, it was first discovered that MSCs in bone marrow can differentiate into bone [6]. Subsequently, several studies have demonstrated that the intravenous delivery of MSCs results in their migration to the injury site, such as bone or cartilage fracture, myocardial infarction, and ischemic brain damage [7–11].

Moreover, certain studies have also reported that MSCs can be used to treat a series of liver diseases including cirrhosis, liver fibrosis, and hepatic ischemia reperfusion [12–15]. For example, Peng and colleagues found that the levels of albulin (ALB), total bilirubin (TBIL), and prothrombin time (PT) and the model for end-stage liver disease (MELD) score of patients in a MSC transplantation group markedly improved 2-3 weeks after transplantation compared to the control group [12]. However, the mechanism of repair remains unclear. How the MSCs transformed into hepatocytes, by differentiation into or fusion with hepatocytes, or neither, is unknown. The results are still controversial [16–20]. In this study, we will demonstrate that MSCs can both differentiate into and fuse with hepatocytes to repair liver damage. In addition, we verified that the SDF-1/CXCR4 axis plays an important role in promoting MSC migration.

## 2. Materials and Methods

### 2.1. MSC Transplantation to Generate a Chimeric Mouse Model

Eight-week-old female wild type C57BL/6 mice were obtained from the Third Military Medical University. The animals were housed in a temperature- and humidity-controlled environment with a 12 h light/12 h dark cycle with food (standard laboratory chow) and water available ad libitum. All animal experiments were approved by the Animal Care and Use Committee of the Third Military Medical University and were performed in compliance with the “Guide for the Care and Use of Laboratory Animals” published by the National Institutes of Health.

To determine if MSCs can differentiate into hepatocyte-like cells, we constructed a chimeric mouse model as previously reported [21]. In brief, MSCs were first dissociated from wild type male C57BL/6 mice. Then, the MSCs were injected into the tail veins of female recipient mice, which had been exposed to a 10 Gy whole-body irradiation using a Co^60^ source (Theratron-780 model; MDS Nordion, Ottawa, ON, Canada).

To determine if MSCs can fuse with hepatocytes, we constructed another chimeric mouse model. In brief, the MSCs were dissociated from GFP^+^ transgenic female C57BL/6 mice and then injected into the irradiated male recipient mice, which had been exposed to a 10 Gy whole-body irradiation. After 20 days, qRT-PCR with peripheral blood cells was used to confirm the chimeric mouse. The process was performed as previously described [22, 23].

### 2.2. Construction of the Acute Liver Damage Model

An acute liver damage model was established 20 days after MSC transplantation as previously described [24]. The mice were injected with CCl_4_ (Sigma, USA) in the abdominal cavity at a dose of 15 *μ*L/g body weight of 0.3%, 0.6%, 0.8%, or 1% CCl_4_ dissolved in peanut oil (Shandong Luhua Group, China). The degree of liver damage was confirmed by histology. On days 2, 3, 4, 7, 14, 21, and 28, damaged liver tissues were collected, and a portion was used for fluorescence in situ hybridization (FISH) and immunofluorescence (IF). Another portion was used for Enzyme-Linked Immune Sorbent Assay (ELISA) to detect the changes in cytokines.

### 2.3. Double Staining by FISH and IF

To study whether exogenous MSCs can differentiate into hepatocyte-like cells or fuse with hepatocytes, we used double staining by FISH and IF analysis as previously described by Luo et al. [25]. In brief, the tissue sections were incubated with the primary anti-ALB monoclonal antibody (1 : 50 Santa Cruz, USA) or anti-GFP monoclonal antibody (1 : 50 Santa Cruz, USA) at 4°C overnight. On the second day, DNA of the Y chromosome was denatured, and the hybridization process was performed following the manufacturer's protocol (StarFISH Cambio, England). The break-apart probe set included DNA fragments against the Y chromosome probes labeled with Cy3 (Cambio, Dry Drayton, UK). On the third day, the slides were washed to remove unbound DNA sequences. Then, the sections were incubated with the secondary antibody of FITC-labeled goat anti-rat IgG (1 : 50 Invitrogen, USA). After washing, the slides were mounted in 40, 6-diamidino-2-phenylindole (DAPI; Invitrogen, Carlsbad, CA), which specifically stains nuclei. Digital images were captured using a confocal laser-scanning microscope with appropriate filters (Leica Biosystems, Wetzlar, Germany) in ten random fields. Experiments were performed in triplicate.

### 2.4. ELISA

The liver tissues collected on days 0, 2, 3, 4, 7, 14, 21, and 28 after injury were lysed as preciously described [26]. ELISA analysis was used to assess the expression of SDF-1 and CXCR4 in the lysates. Analysis was performed following the manufacturer's protocol for the Duoset ELISA Development kit (R&D Systems, Minneapolis, MN). Optical density (OD) values were read on the Synergy microplate reader (Biotek, Winooski, VT) at 450 nm, and a standard curve was constructed using the provided standards for the quantification of ODs for individual samples.

### 2.5. *In Vivo* Chemotaxis Analysis

To analyze the role of SDF-1 in the migration of MSC, 1 × 10^6^ MSCs were preincubated with the SDF-1 inhibitor 17-AAG (Cayman Chemical, Ann Arbor, MI; 20 ng/mL) or PBS for 30 min and injected into the tail veins of bone marrow-destroyed female C57BL/6 mice [27]. The ALI model was created using previously reported methods [24]. FISH and IF were conducted at day 21 after injury.

### 2.6. Statistical Analysis

50 mice were included into these experiments; each group contains at least 3 mice. 5 slices were randomly selected for all the samples and 5 pictures were randomly taken for every slice. Three authors counted the number of both positive cells and total cells. The data are expressed as the mean ± SEM. Student's paired *t*-test was performed for the comparison of data of paired samples, analysis of variance was used for multiple group comparisons, and a Bonferroni posttest was used to determine differences between groups. For all analyses, differences were considered significant at *P* < 0.05. All statistical analyses were performed using the Statistical Program for Social Sciences 13.0 software program (SPSS Inc., Chicago, IL).

## 3. Results

### 3.1. MSCs Were Recruited into Injured Tissue and Transdifferentiated into Hepatocyte-Like Cells

MSCs isolated from male C57BL/6 mice were generously provided by Dr. Guangping Liang (Institute of Burn Research, Third Military Medical University, China). These MSCs were identified as CD44, CD29, and SCA-1 positive but CD117 negative by flow cytometry analyses and were capable of differentiating into adipocytes, chondrocytes, and osteoblasts in vitro [21, 28]. Twenty days after transplantation with these MSCs, the mice were injected with CCl_4_ in the abdominal cavity. We determined that 0.3% CCl_4_ induced the most damage with a large area of cell degeneration and necrosis. Thus, in subsequent experiments we injected 0.3% CCl_4_ into the abdominal cavity to construct the ALI model.

**Figure 1:**
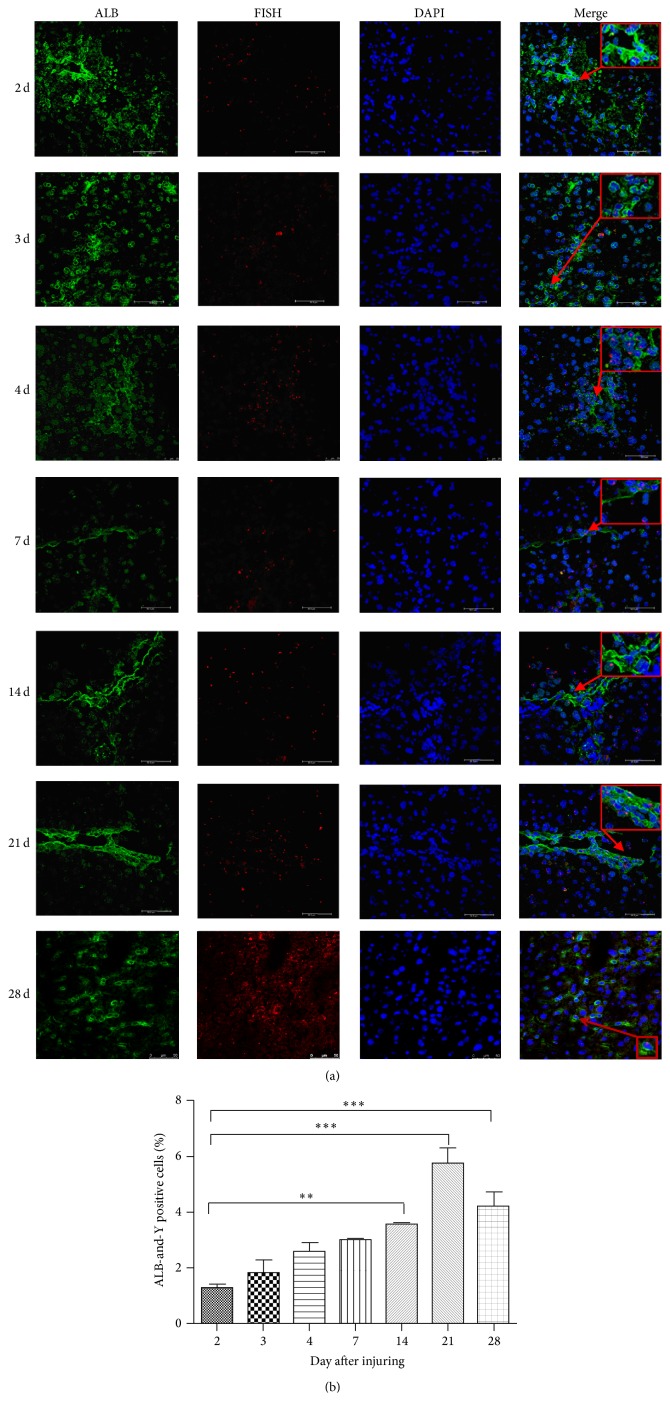
MSCs recruited to injured tissue and transdifferentiated into hepatocyte-like cells. (a) The liver specimens were collected on days 2, 3, 4, 7, 14, 21, and 28 after injury. FISH was used to detect Y-chromosome-positive cells, and IF was used to detect ALB-positive cells. The nuclei were stained blue (DAPI). The images were then merged. Bar: 75 *μ*m. Arrow was directed to the double positive cells. All the experiments were repeated three times, at least 3 samples were included in each group. (b) Quantitative data of the ALB and Y-chromosome-positive cells in IHC of different days after injuring (^**^ *P* < 0.01; ^***^ *P* < 0.001).

To determine if MSCs can transdifferentiate into hepatocyte-like cells, on days 2, 3, 4, 7, 14, 21, and 28 after CCl_4_ injection into chimeric mice, liver specimens were harvested. Because Y-chromosome-positive cells were from the male mice, which represent the MSCs, and ALB is a specific marker for hepatocytes, we used FISH to detect the Y-chromosome-positive cells and IF to detect the ALB-positive cells. As shown in Figure 1(a), expression of the Y chromosome and ALB were detected in the same cells of the damaged liver using laser confocal microscopy. The population of Y-chromosome-positive cells ranged from 13.96% to 18.13%, while the population of both Y-chromosome- and ALB-positive cells ranged from 3.37% to 5.85% (Figure 1(b)). These results show that MSCs can differentiate to hepatocytes.

### 3.2. MSCs Facilitated Liver Repair Partly through Fusion with Primary Hepatocytes

To determine if MSCs facilitated liver repair through fusion with primary hepatocytes, we constructed a male chimeric mouse model as previously described. In brief, the MSCs were extracted from GFP transgenic female mice, which resulted in the GFP labeling of MSCs in the chimeric mouse model.

**Figure 2:**
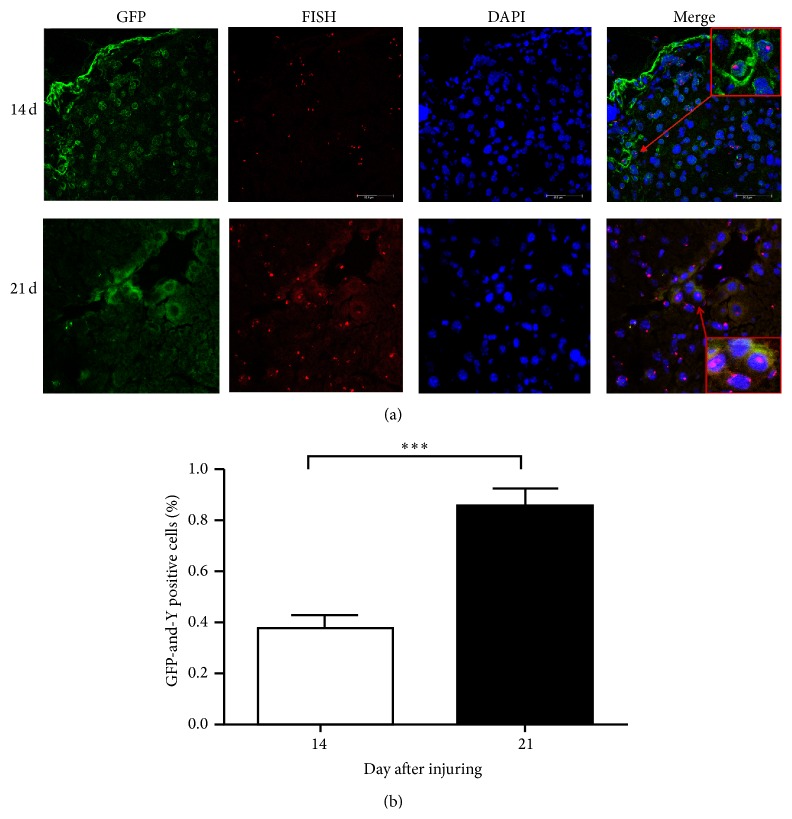
MSCs recruited to injured tissue and fused with hepatocytes. (a) The liver specimens were collected on days 14 and 21 after injury. FISH was used to detect Y-chromosome-positive cells, and IF was used to detect GFP-positive cells. The nuclei were stained blue (DAPI). The images were then merged. Bar: 75 *μ*m. Arrow was directed to the double positive cells. All the experiments were repeated three times, at least 3 samples were included in each group. (b) Quantitative data of the GFP and Y-chromosome-positive cells in IHC of different days after injuring (^***^ *P* < 0.001).

**Figure 3:**
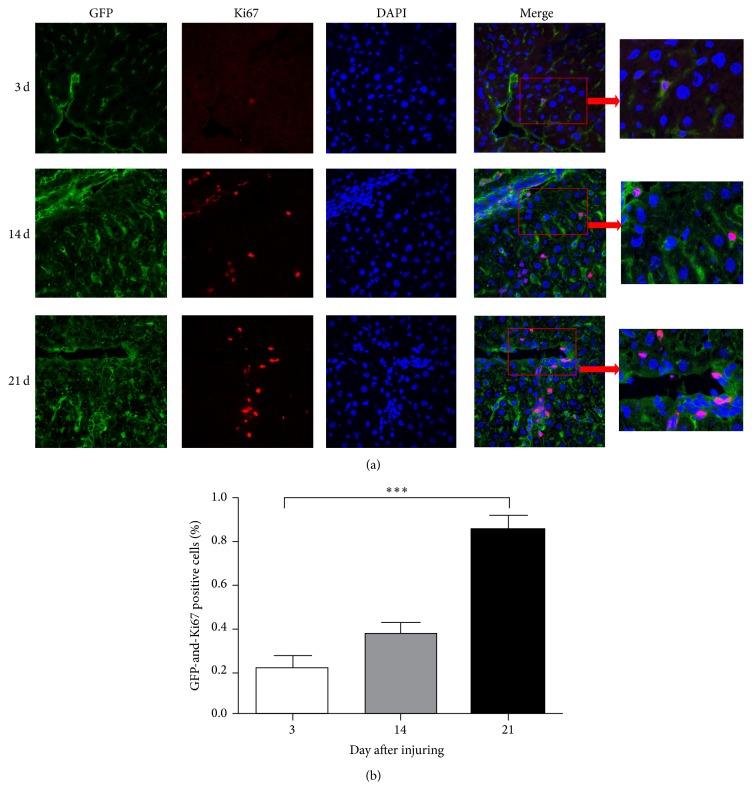
MSCs migrated to the damaged liver and had the proliferative activity. (a) IF analysis of Ki-67 expression (red) to visualize primary hepatocyte cells indicated that some of the donor GFP^+^ MSCs (green) are overlapped. Nuclear DAPI (blue) staining was used as a counterstain. The images were then merged. Bar: 75 *μ*m. Arrow was directed to the double positive cells. All the experiments were repeated three times, at least 3 samples were included in each group. (b) Quantitative data of the GFP and Ki67 positive cells in IHC of different days after injuring (^***^ *P* < 0.001).

On days 14 and 21 after CCl_4_ injection, FISH and IF were used to detect Y-chromosome- and GFP-positive cells. As shown in Figure 2(a), expression of the Y chromosome and GFP could be detected in the same cells of the damaged liver using laser confocal microscopy, which suggests that MSCs can fuse with primary hepatocytes. The population of both Y-chromosome- and GFP-positive cells ranged from 0.32% to 0.87% (Figure 2(b)). In addition, we also detected GFP- and Ki67-positive cells with FISH and IF on days 3, 14, and 21. GFP and Ki67 were expressed in the same cells of the damaged liver, which suggests that the MSCs that migrated to the liver have proliferative activity (Figures 3(a) and 3(b)). Together, these results show that MSCs can fuse with hepatocytes and maintain proliferative activity.

### 3.3. SDF-1/CXCR4 Axis Plays an Important Role in MSC Migration to the Injured Liver

**Figure 4:**
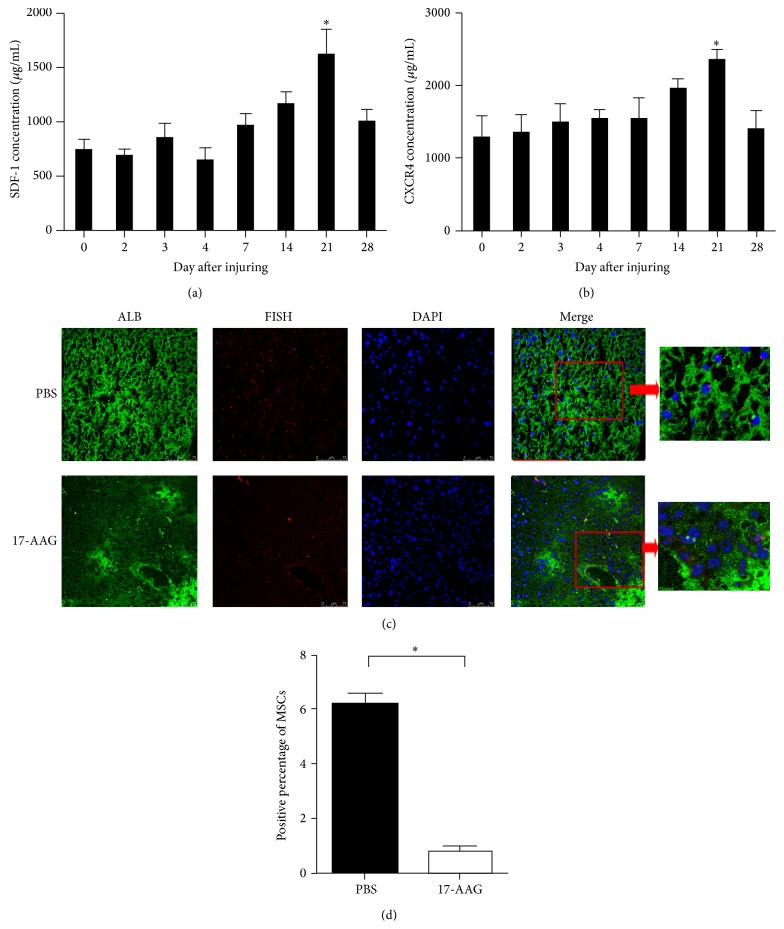
The CXCL12-CXCR4 axis plays an important role in the chemotaxis of MSCs. (a-b) ELISA was used to detect SDF-1 and CXCR4 protein levels in the damaged liver tissue at different times after injury. The data are the mean ± SEM from three independent experiments (^*^ *P* < 0.05 compared with day 0). (c-d) 1 × 10^6^ MSCs were preincubated with 17-AAG (SDF-1 inhibitor, 20 ng/mL) or PBS for 30 min and injected into the tail veins of bone marrow-destroyed female C57BL/6 mice. Then, liver damage was induced in the mice, and tissue sections were collected 21 days after injury. (c) IF was used to detect ALB-positive cells, and FISH was used to identify the Y chromosome-positive cells. Bar: 75 *μ*m. Arrow was directed to the double positive cells. (d) Both ALB- and FISH-positive cells were counted. All the experiments were repeated three times, at least 3 samples were included in each group.

Recent studies have reported that the chemokine SDF-1 and its receptor CXCR4 play a pivotal role in the migration, chemotaxis, homing, and transdifferentiation of MSCs [20]. Therefore, we determined the concentration of SDF-1 and CXCR4 on days 0, 2, 3, 4, 7, 14, 21, and 28 by ELLISA. As shown in Figure 4(a), the concentration of SDF-1 gradually increased and reached its peak on day 21. Consistent with SDF-1, the expression of CXCR4 was also significantly elevated on day 21 (Figure 4(b)).

To further study the role of SDF-1/CXCR4 axis in the migration of MSCs, the female chimeric mice were divided into two groups. One group was injected with MSCs treated with the SDF-1 inhibitor 17-AAG, and the other group was injected with MSCs treated with PBS. On day 21, liver specimens were harvested and analyzed using FISH and IF. As shown in Figures 4(c) and 4(d), Y-chromosome- and ALB-positive cells were notably reduced in the 17-AAG group compared to the PBS group, which suggests that 17-AAG inhibited the migration of MSCs to the damaged liver.

## 4. Discussion

In 1999, Petersen and colleagues found that BM-derived cells may act as the progenitor of several types of liver cells under certain physiopathological conditions [29]. Shortly after that, Theise et al. also reported similar results in humans [30]. Liver specimens were obtained from 2 female recipients of therapeutic bone marrow transplants with male donors and from 4 male recipients of orthotopic liver transplants from female donors. Using FISH and diaminobenzidine (DAB) stain, it was found that Y-positive hepatocytes and cholangiocytes could be identified in all study specimens, which suggested that human hepatocytes and cholangiocytes can be derived from extrahepatic circulating stem cells, most likely of bone marrow origin [30]. However, it was still unclear how MSCs changed into hepatocytes. Recently, certain studies have demonstrated that MSCs have the ability to differentiate into cells with hepatocyte-like phenotypes [31–33]. For example, Sato and colleagues had found that MSCs were more potent than CD34^+^ cells and non-MSCs/CD34^−^ cells in differentiating into hepatocytes [31]. These results were consistent with our findings. In the female chimeric mouse model, MSCs could differentiate into hepatocytes and were able to secrete albumin (Figure 1). In addition, using a male chimeric mouse model, it was found that MSCs could also fuse with hepatocytes to repair liver damage (Figure 2). However, these results apparently contradicted those by Sato, who showed that no evidence was found for MSCs fusing with hepatocytes [31]. This contradiction may be a result of Sato using human MSCs in rat model, which may lead to interspecies hybrids during cell division. Interspecies hybrids will “kick out” or loosen chromosomes of one or the other species because the chromosome complement of interspecies hybrids is not stable [34]. In addition, we also found that the differentiation rate was significantly higher than that of fusion rate, which suggests that differentiation rather than fusion is the main pathway for repairing the damaged liver.

Chemokines play important roles in controlling cell migration. It has been reported that the SDF-1/CXCR4 axis is constitutively expressed in a wide range of tissues such as the brain, heart, kidney, liver, lung, and spleen and also involved in several diseases such as rheumatoid arthritis, ischemic cardiomyopathy, and several brain diseases [35–37]. Moreover, studies have demonstrated that the interaction of SDF-1 with its receptor CXCR4 plays a role in mediating MSC migration to the site of injury [38, 39]. Although it is considered that CXCR4 is expressed primarily in the cell rather than on the surface, it has been assumed that the majority of intracellular CXCR4 in MSCs is mobilized to the cell surface during cytokine stimulation [40, 41]. Furthermore, various organs increase the expression of SDF-1 when responding to tissue damage, such as irradiation, hypoxia, or toxic agent exposure [42, 43]. Therefore, we assume that CCl_4_-induced liver injury is a strong promoter of SDF-1 expression and CXCR4 mobilization. Figures 4(a) and 4(b) show an increase in SDF-1 and CXCR4 concentrations at day 7, which peaked at day 21. Notably, as shown in Figures 4(c) and 4(d), the migration of MSCs significantly decreased when treated with the SDF-1 inhibitor 17-AAG. These data demonstrate that the SDF-1/CXCR4 axis plays an important role in MSC migration to the injured liver for repair. Furthermore, recent study also found that MSCs secreted scrapie responsive gene 1 (SCRG1) and its receptor bone marrow stromal cell antigen 1 (BST1) played important roles for cell self-renew and migration [44]. However, it still needs further study to find its role in MSCs treatment.

However, our results cannot completely explain the curative effects of MSCs for ELSD in the clinical context. A recent study reported that despite the role of transdifferentiation and fusion, MSCs also possess another attractive ability for cell-based therapies. An increasing number of studies have shown that MSCs have potent immunosuppressive qualities [45]. English et al. found that MSCs cocultured with purified CD4^+^ T cells result in a significant increase in FoxP3^+^CD25^+^CD4^+^ T cells, while MSC-derived factors such as TGF*β* and PEG2 play important roles during induction [46]. In addition, recent study also found that MSCs secreted VCAM-1 also played important roles to induce immunosuppression environment [47]. So our future studies will focus on how MSCs induce an immunosuppressing microenvironment to repair the damaged liver.

In conclusion, we report that MSCs can repair the damaged liver by differentiating into and fusing with hepatocytes. Among these two, MSCs differentiating into hepatocytes is the main pathway in repairing the damaged liver. Furthermore, we also found that the SDF-1/CXCR4 axis plays an important role in the chemotaxis, homing, and differentiation of MSCs to the injury site.

## Abbreviations

MSCs: Mesenchymal stem cells
ESLD: End-stage liver disease
CCl_4_: Carbon tetrachloride
ALB: Albumin
TBIL: Total bilirubin
PT: Prothrombin time
MELD: Model for end-stage liver disease
SDF-1: Stroma-derived factor-1
CXCR4: Chemokine (C-X-C motif) receptor 4
GFP: Green fluorescent protein
ELISA: Enzyme-linked immunosorbent assay
FISH: Fluorescence in situ hybridization
IF: Immunofluorescence.
